# Health Anxiety and Its Correlations with Self-Perceived Risk and Attitude on COVID-19 among Malaysian Healthcare Workers during the Pandemic

**DOI:** 10.3390/ijerph18094879

**Published:** 2021-05-03

**Authors:** Hajar Mohd Salleh Sahimi, Nazirah Azman, Nik Ruzyanei Nik Jaafar, Tuti Iryani Mohd Daud, Azlin Baharudin, Ahmad Khaldun Ismail, Akramul Zikri Abdul Malek, Mohd Rohaizat Hassan, Azmawati Mohammed Nawi

**Affiliations:** 1 Department of Psychiatry, Hospital Canselor Tuanku Muhriz, Kuala Lumpur 56000, Malaysia; hajarmss@ppukm.ukm.edu.my (H.M.S.S.); drnazirahazman@gmail.com (N.A.); tutimd@ppukm.ukm.edu.my (T.I.M.D.); jelin72@hotmail.com (A.B.); 2 Department of Emergency Medicine, Faculty of Medicine, Universiti Kebangsaan Malaysia, Kuala Lumpur 56000, Malaysia; khaldun_ismail@yahoo.com; 3 Department of Psychiatry and Mental Health, Ministry of Health Malaysia, Kuala Lumpur 50586, Malaysia; drakramresearch@gmail.com; 4 Department of Community Health, Faculty of Medicine, Universiti Kebangsaan Malaysia, Kuala Lumpur 56000, Malaysia; rohaizat@ppukm.ukm.edu.my (M.R.H.); azmawati@ppukm.ukm.edu.my (A.M.N.)

**Keywords:** front liners, COVID 19, health attitude, health risk perception

## Abstract

Healthcare workers (HCW) are exposed to health-related anxiety in times of pandemic as they are considered to have a high risk of being infected whilst being the vital workforce to manage the outbreak. This study determined the factors that influence health anxiety and its extent in correlations with perceived risk, knowledge, attitude, and practice of HCW. A cross-sectional online survey was conducted on a total of 709 HCW from both public and private healthcare facilities who completed a set of questionnaires on sociodemographic data, knowledge, attitude, and practice of HCW on COVID-19, and health anxiety traits assessed using the short version Health Anxiety Inventory (HAI). Multiple linear regression (adjusted R^2^ = 0.06) revealed respondents with higher perceived risk for COVID-19 significantly predicted higher HAI scores (beta 1.281, *p* < 0.001, 95%, CI: 0.64, 1.92), and those with a higher cautious attitude towards COVID-19 significantly predicted higher HAI scores (beta 0.686, *p* < 0.001, 95%CI: 0.35, 1.02). Healthcare workers’ perceived risk and cautious attitude towards COVID-19 might be potentially influenced by management of the sources and approaches to the dissemination of information of the pandemic. The implementation of certain measures that minimize the infection risk and its related anxiety is important to preserve both their physical and psychological wellbeing.

## 1. Introduction

The COVID-19 pandemic is said to be the impetus of a sequelae of mental health issues [1]. While any flu-like symptom increases psychological distress, the impact of the COVID-19 pandemic on mental health was anticipated to be more serious. Among the psychiatric disorders, anxiety and depression are said to be the leading manifestation [2], with a high probability of increased new-onset health-related anxiety [3]. Health anxiety is defined as worries and anxiety due to perceived threat to one’s health. This is conceptualized as a dimensional construct on a continuum that ranged from the absence of health awareness to disordered health anxiety such as illness anxiety disorder [4,5,6]. There were also reports on the emergence of “psychogenic COVID-19” described as a conversion reaction that presented with upper respiratory tract symptoms and psychogenic fever [7], which may be within the continuum of health anxiety.

The pandemic adds an emotional burden to the infected person through its neuropsychiatric sequelae associated with the viral infection and its medical management [8]. Some people who underwent quarantine experienced many psychological effects from trauma, social isolation, and stigma [3]. Anxiety is also reflected in the society as seen from reported public behaviour, such as panic buying and hoarding of essential and non-essential items [9]. The sudden change in lifestyle, including restricted traveling, working or studying at home, social distancing, and self-isolation, may also contribute to the rise of mental health issues [1].

It is hypothesized that the pandemic will have a considerable psychological impact on HCW in particular as they may have perceived themselves to be at highest risk of exposure to infection. This fear of being infected with COVID-19 possibly due to concerns about their own vulnerability in contracting the disease, worries about inadequate medical supply or protective gears, and/or long working hours. This is supported by an early study of COVID-19 cases in China, where a high rate of 3.8% of exposed HCW (1716 of 44,672) were infected [8]. World Health organization reported that 14%–35% of COVID-19 cases reported to WHO were among HCW [9]. In a previous MERS-CoV outbreak, a study reported a significant number of hospital workers in Saudi Arabia had anxiety of acquiring the infection [10]. Similarly, a recent multi-center survey in China involving 1563 medical staff found the prevalence of depression, anxiety, insomnia, and stress-related symptoms to be 50.7%, 44.7%, 36.1%, and 73.4%, respectively during the COVID-19 pandemic [11]. Attention to the psychological health of frontline healthcare workers is therefore crucial, as their clinical services are much needed for the care of sick patients.

In Malaysia, the COVID-19 pandemic led to a nationwide Movement Control Order (MCO) implemented by the government beginning 18 March 2020. It was enforced in phases until it entered a recovery phase starting on 10 June onwards [12]. The worldwide shortage of personal protection equipment (PPE) during this early phase affected Malaysia’s HCW as well, which was an important source of distress. Having accurate knowledge and a positive attitude with the correct practice of managing the risk of infection may be fundamental to good mental health of the HCW. In turn, HCW with good emotional wellbeing are reliable advocators and change agents in their families and communities. Nevertheless, the extent of knowledge, attitude, and practice regarding COVID-19 of the HCW in relation to their tendency to health anxiety has not been explored. This study specifically investigates the correlations between health anxiety traits and knowledge, attitude, and practice of Malaysian healthcare workers (HCW) during the initial phases of the first Movement Control Order (MCO) in Malaysia, i.e., between 18 March and 28 April 2020. This is important to understand further measures that could be implemented to ensure the concordance of physical and mental health wellbeing of the HCW during a pandemic.

## 2. Methods

### 2.1. Study Design, Setting, and Population

This is a cross-sectional study conducted on a sample of healthcare workers working either in government or private healthcare settings in Malaysia via convenience sampling. Malaysia has 61,158 doctors, a total of 106,373 nurses, about 13,420 pharmacists, and a total of 9699 dentists [13]. Malaysia, with a population of approximately 32 million, has a comprehensive healthcare system constituting of both public and private sectors encompassing 154 government hospitals, 210 private hospitals, 1090 government health clinics, and 7718 private clinics. During the pandemic, Malaysia designated 27 ‘COVID hospitals’ to the admission and isolation of patients under investigation and confirmed cases of COVID-19 [14].

This study was done during the initial phases of the Movement Control Order (MCO) in Malaysia between 18 March 2020, and 28 April 2020. The MCO is a measure equivalent to a varying degree of lockdown in other countries. It was announced by the prime minister of Malaysia two days prior to its commencement as a measure of the outbreak control. During this period, there were restrictions towards movement, mass gatherings and entry of foreign visitors and closure of schools, colleges, universities, house of worships, business and premises except for essential industries and services. Healthcare is an essential service that operates under a lot of pressure due to the pandemic.

This study included all types of HCW comprising doctors, pharmacists, dentists, nurses, occupational/physical therapists and assistant medical officer with the following inclusion criteria: (i) aged between 18- and 60-years old and (ii) able to read and write in English or Malay language. Anyone with known anxiety disorders were excluded from the survey analysis. The sample size needed for the study was 139. Using 10% as prevalence rate of health anxiety disorder according to DSM 5 [15]. The survey was sent through the representatives of the professional bodies, i.e., Malaysian Medical Associations and Malaysian Psychiatric Associations, who distributed the form via the electronic medium. It could not be determined how many received the invitation to participate and how many responded and therefore, the response rate could not be established. Each subject consented to participate by agreeing to continue after going through the survey information sheet in the Google Form. Subsequently, they were directed to complete the self-report questionnaire.

### 2.2. Measures

This questionnaire consists of four components: (a) sociodemographic data (b) COVID-19 related information (c) knowledge, attitude and practice of HCW on COVID-19 and (d) health anxiety inventory.

(a) Sociodemographic data: This includes age, gender, ethnicity, marital status, education status, and work characteristics such as type of employment (doctor/nurses/occupational therapists etc), duration of employment, working hours, and employment status (temporary/permanent) and location of employment (Public setting/private setting).
(b) COVID-19 related information: Respondent’s self-perceived COVID-19 infection risk was also obtained. There were no definitions on the perceived risks as they were mainly subjective response of the respondents based on their own perspectives and their personal life circumstances. We also obtained data on received COVID-19 information, sources of information, and trusted channels to receive information regarding COVID-19.
(c) Knowledge, attitude and practices (KAP) on COVID-19: For this study, a 25-item questionnaire in both Malay and English language was adapted from the World Health Organisation (WHO) Risk Communication and Community Engagement Action Plan Guidance COVID-19 preparedness and response [16]. There were eight items on knowledge (e.g., ‘Which group at high risk?’, ‘how does COVID-19 spread?’), nine items on attitude (e.g., ‘Do you think handling infected COVID-19 cases will threaten the safety of healthcare workers?) and three items on practices (e.g., ‘practices to prevent contracting COVID-19’). Participants were given multiple choice answers for each question. For the items on knowledge, a correct response to an item was assigned 1 point while an incorrect response was assigned 0. Altogether, there were a total of 24 points on knowledge items with higher scores indicating better knowledge about COVID-19. As for the attitude items, there were a total of 10 points allocated and they were scored based on the positive attitudes to adopt in line with the government policy in facing COVID-19. The items on practices were scored based on the correct practices if family were infected with COVID-19 and also correct preventive practices with a total score of 10. (please refer to Supplementary Table S1 for each component of the questionnaire with the allocated points)
(d) Health Anxiety Inventory (HAI): For this study, the short version of HAI was used. This screening instrument contains 14 items that assess health anxiety independently of physical health status. Items assess worries about health, awareness of bodily sensations or changes and feared consequences of having an illness. The short version HAI has demonstrated good reliability, criterion validity, and sensitivity to treatment [17]. At this time the short HAI has not been validated locally in Malaysia. This original version was then translated to Malay language by the current research team. The translation process was based on recommendations by the World Health Organisation (WHO) Process of Translation and Adaptation of Instruments guidelines [18,19,20]. Permission for the translation was granted by its author, Professor Paul Salkovski. The translation to Malay was done by a trainee psychiatrist in the research team; then it was back translated to English by a psychiatrist who is a co-researcher of this study. Subsequently, any discrepancies between the original English HAI and the back translated version were compared, resolved, and finalized by two other co-researchers. The internal consistency of the Malay version of HAI has a Cronbach alpha value of 0.879.

## 3. Statistical Analysis

Data from Google Form were extracted, recorded on an Excel sheet document, and coded. The data were then analysed using Statistical Package for Social Sciences version 25 (SPSS) [21] after checking the missing and outlier data. Frequency and percentage were used to describe categorical data, while a mean (standard deviation) was used for continuous data. An independent t-test and one-way ANOVA were used for bivariable analysis. Simple linear regressions were performed to assess significance of each predictor with HAI score. The significant predictors were then included in a multiple linear regression to assess the linear relationship between the significant predictors with HAI score. Multicollinearity and interaction were checked. All the multiple linear regression assumption was fulfilled. The significant *p*-value was set at <0.05.

## 4. Results

Data was collected from HCW in Malaysian healthcare setting who completed the survey. Of 776 samples, 51.5% were doctors, 25.1% were nurses, 1.4% were pharmacists, while the rest were mainly supporting staffs and administrators. About three quarters of the respondents were female (77.4%) while a majority were aged between 31–40 years old (54.4%), Malay (81.1%), married (70.0%), had a degree as their minimum education level (62.6%), and were permanent staffs (96.5%). The majority of HCW reported receiving information on how to protect from COVID-19 (98.4%), how COVID-19 was transmitted (97.7%), information on COVID-19 symptoms (98.6%), what to do if you have symptoms (95.6%) and risk and complication of COVID-19 (93.9%). A high number of respondents received COVID-19 information from the Facebook application (95.1%), other healthcare workers (90.3%), and other social media applications (86.3%). The majority of respondents did not trust Facebook (72.2%) and other social media applications (79.3%) as trusted channels to receive information, and the most trusted source of information was other healthcare workers (83.2%).

Data from thirteen respondents were removed due to more than 30% missing data and sensitivity analysis revealed non-significant difference (*p* = 0.671). Sixty-one respondents reported known to have anxiety disorder, and hence were excluded from the analysis. Total data taken for analysis was 709.

Table 1 illustrates the association between source of information and self-risk perception. From the table, 61.7% respondents received information from the radio have significantly high self-perceived risk. A higher proportion of respondents (58.7%) who had high self-perceived risk did not obtain information from NGO and this association is statistically significant.

Table 2 demonstrates the association between source of information and knowledge, attitude, and practice scores. In terms of knowledge component, those who received information from Facebook, social media, family and friends had significantly higher knowledge score than those who did not. There was no significant difference in knowledge scores between those who sought information from radio, television, healthcare worker, NGO, community, and religious leaders. As for attitude component, those who received information from television had significantly higher attitude score among those who did not. There was no significant difference in attitude scores between those who sought information from other sources of information. As shown in Table 2, those who received information from radio, television, family members, friends, NGO, community leaders and religious leaders had significantly higher practice score compared to those who did not receive from these sources respectively. Those who received information from another healthcare worker had significantly lower practice scores compared to those who did not.

Table 3 presents the mean score of HAI by sociodemographic variables and self-perceived COVID-19 risk. Overall, there is a significant difference between the groups perceived at risk with no perceived risk group (*p* < 0.001, 95% CI means difference: 1.55, 10.23). A difference is also seen between those with high self-perceived COVID-19 risk who had significantly higher mean HAI scores and those with moderate self-perceived COVID-19 risk (*p* = 0.002, 95% CI mean difference: 1.10, 7.58).

As shown in Table 4, simple linear regression revealed the significant predictors of HAI were self-perceived COVID-19 risk and attitude scores based on KAP. In multiple linear regression, high self-perceived risk remained significant. Those who perceived themselves to have high self-perceived COVID-19 risk was associated with an increase in 1.3 HAI score compared to those with low self-perceived COVID-19 risk. Higher scores in attitude component in KAP reflects a greater cautious attitude towards COVID-19. Each additional attitude score is significantly associated with an increase of 0.7 HAI score. The predictors measured in this study were able to explain 6% of the HAI score (adjusted R^2^ = 0.06).

## 5. Discussion

Past epidemics such as SARS in 2003 [22], H1N1 in 2009/2010 [23], and Ebola in 2014/2016 [24] have revealed that health-related anxiety and safety behavior are pervasive. To the best of the authors’ knowledge, there is no local research done specifically on health anxiety during the pandemic, although local studies reported anxiety among healthcare workers during the COVID-19 outbreak [25,26,27,28]. Healthcare workers’ self-perception of risk and their attitude towards the infectious disease contribute to health anxiety, as demonstrated by this study. We found that self-perceived high risk of getting infected with COVID-19 and a cautious attitude towards the infection significantly correlated with health anxiety traits.

Overestimation of the threat posed by a viral pandemic has been linked with increased anxiety [29,30]. This is also established in this study as the healthcare workers’ self-perceived risk independently and positively correlated with health anxiety. Self-perception of having high risk might be counterproductive and made worse by increasing work demand, moreover with a sudden surge of cases during a pandemic. Particularly for HCW, they presumably have a greater risk of direct or indirect exposure with the virus than the general public. This impression could lead to perceiving the situation as more real and might amplify their risk perceptions, thus increasing their health anxiety traits. This is supported by [31] as they studied across ten countries and found that people who have had personal and direct experience with the infection would have significantly higher risk perception. It is also important to consider the background of the study period, which was during the early phase of MCO. During this period, the cases were peaking and therefore this would also contribute to the participants’ health anxiety. This study was conducted from 18 March 2020, which coincided with the beginning of the virus outbreak in Malaysia where an accumulation of 790 cases with 2 death were reported. COVID-19 cases started to double from 238 cases on 14 March 2020 to 428 cases on 15 March 2020 and continued to increase exponentially since then. The highest daily COVID-19 positive cases were 190 cases on 15 March 2020 [32]. Consideration of the timeframe of the study is essential to better understand the development of anxiety during the pandemic. It helps to identify its potential resilience and preventive factors in accordance to the development of the pandemic [33]. A study during different stages of the SARS epidemic showed that risk-specific worries significantly associated with compliance to protective behaviours at different stages of the epidemic, but cognitive risk appraisal may inform individual protective behavior later in the epidemic trajectory [34].

In this era, one important factor that highly influences people’s knowledge, attitude, and risk appraisal of pandemics is the internet. As reported in this study, social media was among the most sought-after sources of information, as 94.3% and 85.3% of the subjects obtained information from Facebook and other social media apps, respectively, besides relying on other HCW (89.4%). The social media has a role in precipitating and perpetuating anxiety as observed by way of excessive COVID-19-related Internet use as a form of safety-seeking behavior [35]. This occurs by many ways, whether in the form of misinterpretation, distorted information, dissemination or even fabrication of information on the internet, which could further enhance the distressing safety-seeking behavior [36,37]. Conversely, accurate perceptions of personal and societal risk factors importantly contribute to the success of measures and policies placed to flatten the epidemic curve during a pandemic [37]. As outlined in the protection motivation theory [38,39], threat appraisal and risk perception are determinants of the public’s willingness to cooperate and adopt health-protective behaviors during pandemics, including sanitization practices, physical distancing, and wearing face masks [40,41,42].

Interestingly, although respondents reported receiving the majority of information from social media, the majority also reported not trusting social media as a source of COVID-19 information. A possible explanation is that the majority of respondents are highly educated with a health educational background; they are most likely aware of the trustworthiness of social media as a source of medical information for COVID-19. However, because social media is currently so integrated in our lifestyle, it allows faster and easier spread of unconfirmed or false information. Therefore, it becomes challenging to sift through the abundance of information. In addition, due to the unprecedented nature of the COVID-19 pandemic, a lot of information is still unknown. Hence, it is difficult to judge which information is true or false. The social media ‘infodemic’ has been linked to trigger panic and anxiety [43,44]. Nonetheless, it was information sourced from the radio that was found to be significantly related to high self-perceived risk. A recent European survey reported that broadcast media, which includes radio and television, continues to be the most credible media source in contrast to social media [45]. The same survey has also listed the latter as the least trustworthy, as concurred by this study finding.

It is also noteworthy to observe the different source of information influenced the knowledge, attitude and practice scores of the HCW. Multiple sources of information seemed to influence mostly the practice, followed by knowledge, while attitude was only significantly singly linked to television source (Table 2).

While there is individual variation in people’s stress responses to outbreaks, collectively [46], people’s behavior can influence and modify its contagion [47,48,49]. Therefore, the adoption of certain attitude, specifically one that is parallel to the government policy may influence health anxiety as a manifestation of stress response. In this study, the attitude scale examined the respondents’ viewpoint or outlook on the pandemic. It constitutes of their own personal opinion on the dangerousness of COVID-19, its social implications including stigma against certain groups including HCW as a high-risk group, protective measures employed by themselves, their workplace, and the government in general, perceived risk to themselves and family members, and whether they think they were in line or against the government measures (Refer Supplementary Table).

This study demonstrated that those with higher cautious attitude score had higher traits of health anxiety and attitude was not greatly influenced by the source of information except for broadcast media ie. television. Again, adoption of the “right” attitude is influenced by multiple factors, including the availability, dissemination, accuracy, and interpretation of information about the pandemic, risk perception, and individual and societal characteristics. A few of the questions in the Attitude scale such as ‘Do you think COVID-19 is dangerous?’, ‘Do you think handling infected COVID-19 cases will threaten the safety of healthcare workers?’ are ‘anxiety related’, but crucial in assessing one’s attitude on the pandemic. Harper et al. reported that fear at an early stage of pandemic has a functional role, which increases compliance to public health recommendations in order to prevent further transmissions of the disease. Health anxiety has been conceptualized as existing on a spectrum [3]. Hence, the extent of anxiety may determine the performance whether one can perform optimally, underperform, or experience burn-out [50].

The strengths of this study include that it examined health anxiety traits in a sample population where those with pre-existing anxiety disorders were excluded. Therefore, the findings were more specific on the threat of covid-19 to healthcare workers’ psychological health. It also sampled on a heterogeneous study population whereby the findings could be implied for a wider generalizability. There are several limitations of the study. Firstly, it is not representative of all Malaysian HCW. There was overrepresentation of Malay ethnicity; not the typical expected proportion of ethnicity group as among the Malaysian citizens, Malays constituted 63%; followed by other ethnic groups such as Chinese (24%) and Indians (7%) [51]. Female respondents were also overrepresented in this study. That is, despite the fact the Malaysian population was constituted 16.8 million as compared to 15.8 million females [51]. Secondly, the content validity, internal consistency, and/or test-retest reliability of the “knowledge, attitude and practices (KAP) on COVID-19” measure were not assessed, thus could be a potential source of misclassification bias. Thirdly, many confounding factors including presence of other health problems and their own traumatic experience dealing with pandemic, were not incorporated in this study. The researchers also need to ensure that the survey length was appropriate for the participants to complete to ensure genuine responses and prevent fatigue effects of answering a lengthy survey. Fourthly, we could not determine the response rate of the survey as it was sent through the representatives of the professional bodies who then distributed the form via online whereby the numbers of subjects who received the invitation to participate were not reported. Finally, the online platform of the survey also meant that it is biased towards those who were more internet-proficient.

## 6. Implication

High health anxiety traits among the healthcare workers might exhaust essential human resources during a pandemic. It is important to ensure accurate self-risk assessment and adoption of the “right” attitude in coping with the pandemic. Considerations of certain measures, including approaches to the dissemination of information regarding the pandemic and the implementation of certain standard operating procedures that place structure and organize flow to ensure the healthcare workers’ wellbeing, have to be emphasized during pandemic.

## 7. Conclusions

In conclusion, radio as a source of COVID-19 information is associated with higher self-perceived risk. Those receiving COVID-19 information from Facebook, social media, family members and friends had higher knowledge scores. Those receiving information from television had higher attitude scores, while all sources of information except for Facebook and social media were associated with high practice scores. Both risk perception and attitude influence health anxiety traits among healthcare workers.

## Figures and Tables

**Table 1:** Association between source of information and self-risk perception.

Source of Information	Self-Risk Perception				*p* Value
	High n (%)	Moderate n (%)	Low n (%)	No n (%)	
Radio					
Yes	261 (61.7)	123 (29.1)	37 (8.7)	2 (0.5)	**0.021**
No	146 (51.0)	107 (37.4)	33 (11.5)	0 (0.0)	
Television					
Yes	347 (59.5)	177 (30.4)	57 (9.8)	2 (0.3)	0.060
No	60 (47.6)	53 (42.1)	13 (10.3)	0 (0.0)	
Facebook					
Yes	388 (57.6)	220 (32.6)	64 (9.5)	2 (0.3)	0.511
No	19 (54.3)	10 (28.6)	6 (17.1)	0 (0.0)	
Social media					
Yes	349 (57.0)	202 (33.0)	59 (9.6)	2 (0.3)	0.773
No	58 (59.8)	28 (28.9)	11 (11.3)	0 (0.0)	
Health care worker					
Yes	407 (59.1)	230 (31.1)	70 (9.5)	2 (0.3)	0.050
No	29 (42.0)	31 (44.9)	9 (13.0)	0 (0.0)	
Family members					
Yes	186 (56.9)	104 (31.8)	35 (10.7)	0.6	0.413
No	221 (57.9)	126 (33.0)	35 (9.2)	0 (0.0)	
Friends					
Yes	253 (59.4)	132 (31.0)	39 (9.2)	2 (0.5)	0.346
No	154 (54.4)	98 (34.6)	31 (11.0)	0 (0.0)	
NGO					
Yes	90 (53.3)	57 (33.7)	20 (11.8)	2 (1.2)	0.045
No	317 (58.7)	173 (32.0)	50 (9.3)	0 (0.0)	
Community leaders					
Yes	82 (56.9)	45 (31.3)	16 (11.1)	1 (0.7)	0.693
No	325 (57.5)	185 (32.7)	52 (9.6)	1 (0.2)	
Religious leaders					
Yes	60 (54.5)	35 (31.8)	14 (12.7)	1 (0.9)	0.378
No	347 (57.9)	195 (32.6)	56 (9.3)	1 (0.2)	

**Table 2:** Association between source of information and knowledge, attitude and practice scores.

Source of Information	Knowledge Score Mean (SD)	*p* Value	Attitude Score Mean (SD)	*p* Value	Practices Score Mean (SD)	*p* Value
Radio						
Yes	20.7 (2.237)	0.339	6.82 (1.239)	0.112	9.26 (0.939)	**0.000**
No	20.52 (2.469)		6.66 (1.340)		8.93 (1.111)	
Television						
Yes	20.62 (2.309)	0.857	6.81 (1.265)	**0.023**	9.19 (0.987)	**0.004**
No	20.66 (2.453)		6.52 (1.337)		8.87 (1.148)	
Facebook						
Yes	20.69 (2.263)	**0.016**	6.78 (1.274)	0.067	9.13 (1.025)	0.805
No	19.31 (3.188)		6.37 (1.395)		9.17 (1.014)	
Social media						
Yes	20.75 (2.266)	**0.001**	6.78 (1.267)	0.366	9.16 (1.011)	0.077
No	19.87 (2.609)		6.65 (1.377)		8.96 (1.089)	
Health care worker						
Yes	20.67 (2.209)	0.249	6.78 (1.263)	0.156	6.78 (1.263)	**0.028**
No	20.20 (3.261)		6.55 (1.440)		8.77 (1.447)	
Family members						
Yes	20.83 (2.094)	**0.024**	6.81 (1.221)	0.291	9.26 (0.909)	**0.001**
No	20.45 (2.509)		6.71 (1.332)		9.02 (1.101)	
Friends						
Yes	20.92 (2.150)	**0.000**	6.77 (1.237)	0.823	9.26 (0.880)	**0.000**
No	20.19 (2.526)		6.75 (1.350)		8.94 (1.185)	
NGO						
Yes	20.87 (2.063)	0.118	6.85 (1.263)	0.311	9.37 (0.738)	**0.000**
No	20.55 (2.408)		6.73 (1.288)		9.05 (1.087)	
Community leaders						
Yes	20.93 (2.027)	0.052	6.85 (1.202)	0.354	9.39 (0.720)	**0.000**
No	20.55 (2.401)		6.74 (1.302)		9.06 (1.078)	
Religious leaders						
Yes	20.92 (1.940)	0.099	6.90 (1.141)	0.170	9.37 (0.765)	**0.001**
No	20.57 (2.396)		6.73 (1.306)		9.09 (1.059)	

**Table 3:** Bivariate analysis of sociodemographic, work characteristics, self-perceived risk and Health Anxiety Inventory mean score.

Respondents Characteristics	HAI Score Mean (Sd)	*p* Value
*Demographic*		
**Age group (years)**		0.793
18–30	25.24 (5.54)	
31–40	24.50 (5.28)	
41–50	24.55 (5.28)	
51–60	24.71 (6.21)	
**Gender**		0.758
Male	25.12 (0.46)	
Female	24.91 (5.70)	
**Ethnic group**		0.104
Malay	25.12 (5.68)	
Non Malay	24.23 (5.72)	
**Marriage status**		0.810
Single	25.15 (5.92)	
Married	24.87 (5.70)	
Ever married	25.38 (4.51)	
Education status		0.053
Lower	25.54 (5.73)	
High (Degree & above)	24.61 (5.70)	
**Occupational status**		
Working status		0.082
Temporary	22.32 (4.71)	
Permanent	25.03 (5.73)	
Working hours		0.371
<50 h	25.06 (5.68)	
≥50 h	24.60 (5.82)	
Working duration		0.599
<10 years	25.05 (5.99)	
≥10 years	24.84 (5.40)	
**Perceived COVID-19 risk**		**<0.001** ^a^
High risk	25.96 (5.89)	
Moderate Risk	23.49 (4.59)	
Low Risk	24.08 (6.47)	
No Risk	16.00 (2.83)	

^a^ High risk vs. moderate risk (*p* = 0.002).

**Table 4:** Predictors of HAI score using simple and multiple linear regression (N = 763).

Respondents Characteristics	SLR		MLR		
	Crude B	*p*-Value	Adjusted B	95%CI	*p*-Value
**Demographic**					
Age group (years)	0.019	0.899	-	-	-
Ethnic group (non-Malay vs. Malay)	−0.358	0.529	-	-	-
Marital status (married vs. single)	−0.469	0.331	-	-	-
Education status (high vs. low)	−0.104	0.834	-	-	-
**Occupational status**					
Working status (permanent vs. temporary)	2.200	0.094	-	-	-
Working hours (≥50 h vs <50 h)	−0.420	0.417	-	-	-
**Self-perceived risk, Knowledge, Attitude and Practice score**					
Self-Perceived COVID-19 risk *	1.322	**<0.001**	1.281	0.64, 1.92	<0.001
Knowledge score	0.146	0.116	-	-	-
Attitude score	0.683	**<0.001**	0.686	0.35, 1.02	<0.001
Practice score	−0.181	0.401	-	-	-

SLR Simple linear regression, MLR Multiple linear regression (adjusted to demographic, occupational status self-perceived risk, knowledge, attitude, practice score), CI Confidence interval, adjusted R^2^ = 0.06.

## Supplementary Materials

The following are available online at https://www.mdpi.com/article/10.3390/ijerph18094879/s1, Title: KAP QUESTIONNAIRE (25 ITEMS).
