# Supplementary material for: Health Anxiety and Its Correlations with Self-Perceived Risk and Attitude on COVID-19 among Malaysian Healthcare Workers during the Pandemic
Source: Int J Environ Res Public Health. 2021 May 3;18(9):4879. doi: 10.3390/ijerph18094879 (PMC8124576; doi:10.3390/ijerph18094879)
Supplement: Supplementary file 1 [file ijerph-18-04879-s001.zip › ijerph-1196225-supplementary.pdf]

KAP QUESTIONNAIRE (25 ITEMS)

COMPONENTS	NO OF QUESTIONS	TOTAL MARKS ALLOCATED
Knowledge	8	24 points
Attitude	9	10 points
Practice	3	10 points

Q1. What kind of information have you received about the disease?<br><br>Apakah jenis maklumat yang telah anda terima mengenai penyakit ini?<br><br>a. How to protect yourself from the disease?<br>Bagaimana melundungi diri anda daripada penyakit tersebut?<br><br>b. Symptoms of the new coronavirus disease<br>Simptom-simptom penyakit Coronavirus<br><br>c. How it is transmitted<br>Bagaimana ia tersebar<br><br>d. What to do if you have the symptoms<br>Apa yang perlu dilakukan sekiranya mempunyai simptom-simptom<br><br>e. Risks and complications<br>Risiko dan komplikasi	No mark allocated

Q2 Where did you hear about COVID 19? Mark all channels that you heard about the disease from)<br><br>Dari mana anda mendengar mengenai COVID 19? (Tandakan semua sumber maklumat yang anda terima berkaitan penyakit tersebut)<br><br>a. Radio<br>b. Television<br>c. Facebook<br>d. Social Media (other than Facebook, ie Instagram, Whatsapp, Twitter etc)<br>e. Health care worker / Staf kesihatan<br>f. Family members / Ahli keluarga<br>g. Friends / Rakan-rakan<br>h. NGO / Badan bukan kerajaan<br>i. Community leaders / Ketua komuniti<br>j. Religious leaders / Pemimpin agama<br>k. Traditional healers / Perawat tradisional	No mark allocated

Q3. Which channel / who do you trust the most to receive information related to COVID 19? (one or more options) Melalui sumber mana anda / Dari siapa yang anda paling percaya maklumat yang anda terima mengenai COVID 19? (Boleh tanda lebih dari satu pilihan)<br><br>a. Radio<br>b. Television<br>c. Facebook<br>d. Social Media (other than Facebook, ie Instagram, Whatsapp, Twitter etc)<br>e. Health care worker / Staf kesihatan<br>f. Family members / Ahli keluarga<br>g. Friends / Rakan-rakan<br>h. NGO / Badan bukan kerajaan<br>i. Community leaders / Ketua komuniti<br>j. Religious leaders / Pemimpin agama<br>k. Traditional healers / Perawat tradisional	No mark allocated

ATTITUDE<br><br>Q4. How dangerous do you think the new coronavirus risk is? (Choose the best answer that matches your belief about the disease)<br>Pada pendapat anda, sejauh manakah tahap bahaya penyakit coronavirus ini? (Pilih jawapan yang paling tepat berkenaan kepercayaan anda terhadap penyakit ini)	2 points allocated
**a. Very dangerous / Sangat berbahaya**	**2 points**
b. More or less dangerous / Agak berbahaya	1 point
c. Is not dangerous / Tidak berbahaya	0 point

KNOWLEDGE<br><br>Q5. Who do you think is at highest risk to get the coronavirus? (Mark all those you think are likely to become sick)<br>Pada pendapat anda, siapakah golongan yang paling berisiko tinggi untuk mendapat penyakit Coronavirus? (Sila tanda kesemua yang anda rasa berisiko tinggi)	1 point allocated
a. Children under 5 years old / Kanak-kanak bawah umur 5 tahun	0 point
b. Adolescents up to 15 years old / Kanak-kanak sehingga umur 15 tahun	0 point
c. Youth / Remaja	0 point
d. Adults / Dewasa	0 point
**e. Elderly Person / Orang tua**	**1 point**
f. Pregnant Woman / Wanita yang mengandung	0 point
g. Healthcare worker / Kakitangan kesihatan	0 point

KNOWLEDGE<br><br>Q6. How does COVID-19 spread? (Mark all the possible ways that you think can cause spread of COVID 19)<br>Bagaimanakan COVID-19 tersebar? (Sila tandakan kesemua cara yang anda rasa boleh menyebabkan penyebaran COVID 19)	3 points allocated
a. Blood transfusion / Pemindahan darah	0 point
**b. Droplets from infected people / Melalui titisan air daripada individu yang telah dijangkiti**	**1 point**
c. Airborne / Melalui udara	0 point
**d. Direct contact with infected people / Bersentuhan dengan individu yang telah dijangkiti**	**1 point**
**e. Touching contaminated objects/surfaces / Menyentuh permukaan yang telah tercemar**	**1 point**
f. Sexual intercourse contact / Melalui hubungan seks	0 point
g. Contact with contaminated animals / Bersentuhan dengan haiwan yang tercemar	0 point
h. Mosquito bites / Gigitan nyamuk	0 point
i. Eating contamined food / Memakan makanan yang tercemar	0 point
j. Drinking unclean water / Meminum air yang tidak bersih	0 point
k. Don't know / Tidak tahu	0 point

KNOWLEDGE<br><br>Q7. What are the symptoms of the disease? (More than 1 answer is acceptable)<br>Apakah simptom-simptom penyakit tersebut? (Lebih dari 1 jawapan dibenarkan)	8 points allocated
**a. Fever / Demam**	**1 point**
**b. Cough / Batuk**	**1 point**
**c. Shortness of breath / Sesak nafas**	**1 point**

**d. Muscle Pain / Sakit otot**	**1 point**
**e. Headache / Sakit kepala**	**1 point**
**f. Diarrhoea / Cirit birit**	**1 point**
**g. Sore throat / Sakit tekak**	**1 point**
**h. Chest pain / Sakit dada**	**1 point**
**i. All of the above / Semua di atas**	**8 point**
j. Don't know / Tidak tahu	0 point

**KNOWLEDGE**<br><br>Q8. Do you know how to prevent it? (one or more options)<br>Adakah anda mengetahui bagaimana untuk mencegah penyakit tersebut? (Lebih dari satu pilihan dibenarkan)	3 points allocated
a. Sleep under the mosquito net<br>Tidur di bawah kelambu	0 point
**b. Wash your hands regularly using alcohol or soap and water**<br>**Sentiasa cuci tangan dengan pencuci berasaskan alkohol atau air dan sabu**	**1 point**
c. Drink only treated water<br>Hanya minum air yang telah dirawat	0 point
**d. Cover your mouth and nose when coughing or sneezing**<br>**Tutup mulut dan hidung sewaktu batuk atau bersin**	**1 point**
**e. Avoid close contact with anyone who has a fever and cough**<br>**Elakkan daripada berada dekat dengan individu yang mempunyai demam dan batuk**	**1 point**
f. Eliminate standing water<br>Membuang air yang tidak mengalir	0 point
g. Cook meat and eggs well<br>Masak daging dan telur dengan sempurna	0 point
h. Avoid unprotected direct contact with live animals and surfaces in contact with animals<br>Elakkan daripada bersentuhan secara terus dengan haiwan yang hidup dan permukaan yang telah bersentuhan dengan haiwan	0 point
i. Don't know / Tidak tahu	0 point

**PRACTICE**<br><br>Q9. What have you and your family done to to prevent becoming sick with COVID 19? (Mark all the steps that you have taken) / Apakah yang telah anda dan keluarga anda lakukan untuk mengelakkan daripada mendapat COVID-19? (Tandakan kesemua langkah yang telah dilakukan)	3 points allocated
**a. Washing hands regularly using alcohol-based cleaner or soap / water**<br>**Sentiasa cuci tangan dengan pencuci berasaskan alkohol atau air dan sabun**	**1 point**
**b. Avoid close contact with anyone who has a fever and cough**<br>**Elakkan daripada berada dekat dengan individu yang mempunyai demam dan batuk**	**1 point**
c. Cook meat and eggs well<br>Masak daging dan telur dengan sempurna	0 point
d. Avoid unprotected direct contact with live animals and surfaces in contact with animals<br>Elakkan daripada bersentuhan secara terus dengan haiwan yang hidup dan permukaan yang telah bersentuhan dengan haiwan	0 point
**e. Covering mouth and nose when coughing or sneezing**<br>**Tutup mulut dan hidung sewaktu batuk atau bersin**	**1 point**
f. Don’t know / Tidak tahu	0 point
g. Eliminate standing water / Membuang air yang tidak mengalir	0 point

**PRACTICE**<br><br>Q10. What to do if you or someone from your family has symptoms of this disease? (Mark all the actions you would take)<br>Apakah yang perlu dilakukan jika anda atau ahli keluarga anda mempunyai simptom-simptom penyakit ini? (Tandakan semua langkah yang akan anda ambil)	2 points allocated
a. I will look for a more experienced relative to advise me on what to do	0 point

Saya akan mencari ahli keluarga yang lebih berpengalaman untuk menasihatkan saya berkenaan apa yang saya perlu lakukan	
**b. I will go to the hospital / health unit**<br>**Saya akan pergi ke hospital / fasiliti kesihatan**	**1 point**
c. I will go to the neighborhood nurse<br>Saya akan berjumpa dengan jururawat komuniti	0 point
d. I will buy medicines at the market<br>Saya akan beli ubat di farmasi	0 point
e. I will look for the traditional healer<br>Saya akan mencari perawat tradisional	0 point
**f. I would stay in quarantine**<br>**Saya akan mengkuarantinkan diri**	**1 point**

**ATTITUDE**<br><br>Q11. Do you think the coronavirus disease is generating stigma against specific people? To whom?<br>Adakah anda merasakan penyakit Coronavirus ini menimbulkan stigma terhadap individu tertentu? Kepada siapa?	1 point allocated
a. Yes (If yes, plese proceed to question 17)<br>Ya (Jika ya, sila ke soalan 17)	0 point
**b. No (Go to section 5 (Quarantine))**<br>**Tidak (Sila ke Bahagian 5 (Kuarantin))**	**1 point**

**STIGMA**<br><br>Q12. (If yes) Which group is being discriminated in your community because of coronavirus?<br>(Jika ya) Kumpulan manakah yang didiskriminasi di komuniti anda disebabkan Coronavirus?	No marks allocated

a. Chinese / Orang China	
b. Tabligh	
c. Italians / Orang Itali	
d. People with positive COVID 19 virus / Individu yang dijangkiti COVID 19	

**KNOWLEDGE**<br><br>Q 13. Why do you think 14 days of self-quarantine is needed after you come back from affected countries?<br>Pada pendapat anda, mengapa tempoh 14 hari kuarantin diri sendiri diperlukan jika anda baru pulang daripada negara-negara yang terjejas?	1 point allocated
**a. It is the incubation period of coronavirus / Ia merupakan tempoh inkubasi Coronavirus**	1 point
b. It is the average recovery period of those with COVID-19 positive / Ia merupakan purata tempoh pulih bagi individu yang dijangkiti COVID-19	0 point
c. I don't know / Saya tidak tahu	0 point

**KNOWLEDGE**<br><br>Q14. Are there any specific medication to treat COVID-19?<br>Adakah terdapat rawatan tertentu untuk COVID-19?	1 point allocated
a. Yes / Ya	0 point
**b. No / Tidak**	**1 point**
c. I don't know / Saya tidak tahu	0 point

**KNOWLEDGE**<br><br>Q15. Are therevaccines to prevent COVID-19?<br>Adakah terdapat vaksin untuk mengelakkan COVID-19?	1 point allocated
a. Yes / Ya	0 point
**b. No / Tidak**	**1 point**
c. I don't know / Saya tidak tahu	0 point

KNOWLEDGE<br><br>Q15. The recommended Personal Protective Equipment (PPE) for healthcare workers when dealing with confirmed cases of COVID 19 are…<br>Berikut merupakan Alat Pelindung Diri untuk kakitangan kesihatan sewaktu berdepan dengan kes-kes yang telah disahkan COVID-19.	6 points allocated
**a. Hand hygiene before and after wearing gloves**<br>**Kebersihan tangan sebelum dan selepas memakai sarung tangan**	**1 point**
**b. Using clean, non sterile long sleeved gown**<br>**Menggunakan gaun lengan panjang yang bersih tetapi tidak steril**	**1 point**
**c. Using N95 mask**<br>**Menggunakan topeng muka N95**	**1 point**
d. Sterile gloves<br>Sarung tangan steril	0 point
**e. Gloves that cover over the cufflinks of the gown**<br>**Sarung tangan yang menutup pergelangan tangan gaun**	**1 point**
**f. Surgical mask**<br>**Topeng muka bedah**	**1 point**
g. Paper mask<br>Topeng muka kertas	0 point
**h. Goggles / face shield**<br>**Kaca mata goggle / Penutup muka**	**1 point**
i. Boots<br>Kasut but	0 point

ATTITUDE<br><br>Q17. Do you think the current protective measures by your workplace is sufficient for preventing COVID-19 infection?<br>Adakah anda merasakan langkah-langkah perlindungan yang diambil di tempat kerja anda sudah mencukupi untuk mengelakkan jangkitan COVID 19?	1 point allocated
**a. Yes / Ya**	**1 point**
b. No / Tidak	0 point
c. I don't know / Saya tidak tahu	0 point

ATTITUDE<br><br>Q18. Do you think the government has taken sufficient measures to manage the COVID-19 outbreak?<br>Adakah anda merasakan pihak kerajaan telah mengambil tindakan sewajarnya untuk mengurus wabak COVID 19?	1 point allocated
**a. Yes / Ya**	**1 point**
b. No / Tidak	0 point
c. I don't know / Saya tidak tahu	0 point

ATTITUDE<br><br>Q19. Are you afraid to go to common places due to fear of getting COVID-19?<br>Adakah anda takut untuk pergi ke tempat umum disebabkan takut mendapat COVID 19?	1 point allocated
**a. Yes / Ya**	**1 point**
b. No / Tidak	0 point

**ATTITUDE**<br><br>Q20. Are closure of schools and work places necessary to control COVID 19?<br>Adakah penutupan sekolah dan tempat kerja perlu untuk mengawal COVID 19?	1 point allocated
**a. Yes / Ya**	**1 point**
b. No / Tidak	0 point
c. I don't know / Saya tidak tahu	0point

**ATTITUDE**<br><br>Q21. Do you think that handling infected COVID-19 case will threaten your safety as healthcare worker?<br>Adakah anda merasakan bahawa sebagai kakitangan kesihatan, menguruskan kes COVID-19 akan menjejaskan keselamatan anda?	1 point allocated
**a. Yes / Ya**	**1 point**
b. No / Tidak	0 point
c. I don't know / Saya tidak tahu	0 point

**ATTITUDE**<br><br>Q22. Are you afraid that one of your family members may be infected with COVID-19 through you?<br>Adakah anda khuatir salah seorang ahli keluarga anda mungkin dijangkiti COVID-19 melalui anda?	1 point allocated
**a. Yes / Ya**	**1 point**
b. No / Tidak	0 point

**ATTITUDE**<br><br>Q23. Are you disturbed by the death cases related to COVID 19?<br>Adakah anda terganggu dengan kes-kes kematian berkaitan COVID-19?	1 point allocated
**a. Yes / Ya**	**1 point**
b. No / Tidak	0point

**PRACTICE**<br><br>Q24. I will practise social distancing during outbreak of COVID by... (More than 1 answer is acceptable)<br>Saya akan mengamalkan penjarakan sosial sewaktu wabak COVID-19 dengan… (lebih dari 1 jawapan diterima)	5 points allocated
**a. Avoid going to social gatherings**<br>**Elakkan daripada menghadiri majlis keraian**	**1 point**
**b. Avoid going to shopping malls**<br>**Elakkan daripada pergi ke pusat beli belah**	**1 point**
**c. Avoid going overseas**<br>**Elakkan daripada pergi ke luar negara**	**1 point**
d. Social distancing does not matter<br>Penjarakan sosial tidak mempunyai makna	0 point
**e. Avoid leaving the house**<br>**Elakkan daripada keluar dari rumah**	**1 point**
**f. Avoid travelling to other states**<br>**Elakkan daripada mengembara ke negeri-negeri lain**	**1 point**

**ATTITUDE**<br><br>Q25. What do you think is your risk status?<br>Apakah pendapat anda berkenaan status risiko anda?	No point allocated
a. High risk / Berisiko tinggi	
b. Moderate Risk / Risiko Sederhana	
c. Low Risk / Risiko Rendah	
d. No Risk / Tiada Risiko	
